# CT-Based Risk Assessment After Left Atrial Appendage Occlusion: Methodological Considerations

**DOI:** 10.1016/j.jacadv.2026.102799

**Published:** 2026-05-14

**Authors:** Kamal Isgandarov, Bulent Gorenek

**Affiliations:** a Özel Bandirma Royal Hastanesi, Bandirma, Turkey; b Eskişehir Osmangazi University, Eskisehir, Turkey

We read with great interest the study by Milutinovic et al^1^ evaluating the potential role of preprocedural and postprocedural cardiac computed tomography (CT) in identifying patients at risk for complications following left atrial appendage occlusion (LAAO). However, several methodological aspects of the study merit further clarification.

First, the authors report a higher frequency of adverse events among patients with larger ostial diameters, areas, and perimeters. These measurements, however, constitute the primary parameters used during LAAO planning to select an appropriately sized device.^2^^,^^3^ Despite significant differences in anatomical measurements between groups, the device sizes used were similar. This finding may suggest that device sizes appropriate for the ostial dimensions may not have been chosen during the procedure.

Second, although an association between the change in device area (ΔA) and adverse events was reported, no clinically applicable threshold value for this parameter was defined. Without establishing a cutoff value or presenting analyses demonstrating discriminatory performance, it remains difficult to conclude that CT imaging can reliably identify patients at higher risk. Moreover, ΔA is derived from postprocedural imaging, at a time when complications such as peridevice leak may already be detectable. In this context, CT imaging appears to reflect existing complications rather than identify patients at higher risk.

Third, the authors suggest that a more oval ostium may facilitate better device seating and potentially reduce peridevice leak formation. However, the literature contains conflicting findings regarding the role of ostial ovality, including studies reporting opposite associations.^4^ Given that preprocedural CT imaging was available in the present study, the relationship between ovality and outcomes could have been directly assessed by calculating an ovality index derived from maximal and minimal diameters. Quantitative evaluation of this parameter might have strengthened the interpretation of the findings beyond a hypothetical explanation.

Finally, the study includes procedures performed between 2022 and 2025; however, the temporal distribution of adverse events during this period was not evaluated. In interventional therapies such as LAAO, operator experience and learning curves are well-recognized determinants of procedural outcomes.^5^ Determining whether complications were more frequent during earlier phases of the study period could help clarify the interpretation of the reported associations.

In conclusion, addressing the aforementioned issues may further clarify the interpretation of the findings and strengthen the contribution of the study to the existing literature.
